# Incidence and risk factors for urinary tract infections in the first year after renal transplantation

**DOI:** 10.1371/journal.pone.0251036

**Published:** 2021-05-03

**Authors:** Arzu Velioglu, Gokhan Guneri, Hakki Arikan, Ebru Asicioglu, Elif Tukenmez Tigen, Yiloren Tanidir, İlker Tinay, Cumhur Yegen, Serhan Tuglular

**Affiliations:** 1 Division of Nephrology, Department of Internal Medicine, Marmara University School of Medicine, Istanbul, Turkey; 2 Department of Infectious Disease, Marmara University School of Medicine, Istanbul, Turkey; 3 Department of Urology, Marmara University School of Medicine, Istanbul, Turkey; 4 Department of General Surgery, Marmara University School of Medicine, Istanbul, Turkey; University of Toledo, UNITED STATES

## Abstract

**Background:**

The most common infections among renal transplant patients are urinary tract infections (UTI). Our main objective in this study is to determine the incidence of UTIs in patients who have undergone renal transplantation in our hospital, to identify the causative microbiological agents, risk factors and determine the effects of UTI on short-term graft survival.

**Methods:**

Urinary tract infections, which developed within the first year of renal transplantation, were investigated. Patients were compared regarding demographic, clinical, laboratory characteristics and graft survival.

**Results:**

102 patients were included in our study. Fifty-three patients (53%) were male and 49 (48%) were female. Sixty-seven urinary tract infection attacks in 21 patients (20.5%) were recorded. Age (p = 0.004; 95% Confidence Interval [CI]: 1.032–1.184), longer indwelling urinary catheter stay time (p = 0.039; 95% Confidence Interval [CI]: 1.013–1.661) and urologic complications (p = 0.006; 95% Confidence Interval [CI]: 0.001–0.320) were found as risk factors for UTI development in the first year of transplantation. *Escherichia coli* and *Klebsiella pneumoniae* were the most frequently isolated microorganisms. Of these bacteria, 63.2% were found to be extended spectrum beta lactamase (ESBL) positive. Multidrug resistant microorganisms (MDROs) were more frequent in male patients (32 episodes in males vs. 14 episodes in females, p = <0.001). UTI had no negative impact on short-term graft survival.

**Conclusion:**

Our study results represent the high incidence of UTI with MDROs in KT recipients. Infection control methods should be applied even more vigorously especially in male transplant patients since a higher incidence of UTI caused by resistant microorganisms was reported in male patients.

## Introduction

In kidney transplant recipients, the risk of infection is increased compared to the general population due to intensive immunosuppressive drug use, surgical procedures and environmental factors. The most common infections in renal transplant patients are urinary tract infections (UTI). The UTI rate in renal transplant patients has been reported to range between 20 to 80% [1–3].

Many different mechanisms and risk factors are responsible for the increased frequency of UTI in renal transplant patients. The most commonly reported risk factors are advanced age, female gender, diabetes mellitus, urinary system abnormality and previous history of urinary system infection. Deceased kidney transplantation, re-transplantation, neurogenic bladder dysfunction, vesicoureteral reflux, and in-dwelling urinary catheters also increase the risk of urinary tract infection. In addition, intensive immunosuppressive use after transplantation also contributes to the increased susceptibility to infections [2, 4, 5].

The microorganisms that cause these infections seem to differ between countries and regions, but the most common causative microorganisms uniformly throughout the world are gram-negative bacteria. These are, in order of frequency, *Escherichia coli* (30–80%), *Klebsiella pneumoniae* (10%), *Proteus* (5%) and *Pseudomonas* (5%) species. Gram-positive bacteria such as *Enterococcus sp*. and *Staphylococcus aureus* are also more common in these patients compared to the general population [6–8]. The increasing prevalence of UTIs caused by multidrug-resistant microorganism (MDRO) is one of the major challenges in transplant patients [6, 9, 10]. The latter situation makes UTI management more complex with the increased need for hospitalization at higher costs.

The frequency of urinary tract infections may also differ between countries and centers considering the environmental, social and economic characteristics. Therefore, determining the frequency and risk factors of urinary tract infections in each transplantation population is very important for determining prevention and treatment strategies.

The aim of our study is to determine the incidence of urinary tract infections among our kidney transplant recipients within the first year of transplantation, to determine the causative microorganisms as well as the underlying risk factors and to determine the impact of UTI on short-term graft survival.

### Patients-methods

This single-center retrospective cohort study was performed at the Marmara University School of Medicine, Transplantation clinic. Patients who underwent renal transplantation between October 2011 and July 2018 were included in the study. Patients younger than 18 years of age and patients with primary non-functioning kidney were excluded from the study. This study complied with the principles of the Declaration of Helsinki and was approved by Institutional Research Ethics Board of Marmara University School of Medicine (approval ID: 09.2017.429). The ethics committee waived the requirement for informed consent due to retrospective nature of the study.

Urinary tract infections that developed in the first year after transplantation were examined retrospectively. Basiliximab was used as standard induction, while anti-thymocyte globulin (ATG) was given to patients at high immunological risk. The patients were started on a triple-drug immunosuppressive regimen typically consisting of calcineurin inhibitors (tacrolimus or cyclosporine), mycophenolate mofetil (MMF), and prednisone. Corticosteroids were progressively tapered to 5 mg/day over 3 months. Induction regimens and maintenance immunosuppressive drugs at follow-up were also recorded.

Antibiotic prophylaxis with single dose cefuroxime at a dose of 2 g was used in all patients before surgery. Ofloxacin 400 mg/day was administered after transplantation for 5–7 days. Trimethoprim/sulfamethoxazole (TMP/SMX) was started for *Pneumocystis jirovecii* prophylaxis on 7th day post transplant and continued for 6 months at a dose of 400/80 mg/day. The ureteral stents placed during the transplant operation were removed at 6–8 weeks after transplantation, in the absence of active UTIs. Post-transplant urologic complications were defined as a need for intermittent catheterization, bladder atony, need for surgical reconstruction of the urinary tract including ureteral necrosis, and ureteral and/or urethral strictures. [11]

UTI was defined as the presence of urine culture positivity with more than 10^5^ colony-forming units (CFU) of bacteria per mL with UTIs symptoms. A positive urine culture without any clinical symptoms was considered as asymptomatic bacteriuria (ABU). According to patients’ clinical presentations, UTI attacks were divided into three groups: Lower UTI (LUTI), Complicated UTI (CUTI) and ABU (1). All UTI patients with ureteral stent were treated. Recurrent UTI was defined as ≥ 3 UTIs in any 12-month period or ≥2 UTIs in any 6-month period, irrespective of the causative organism [12].

Extended-spectrum beta-lactamase-producing organisms (*Escherichia coli*, *Klebsiella pneumoniae*, *Enterobacter spp*., *or Citrobacter spp*.), methicillin-resistant *Staphylococcus aureus*, *Acinetobacter baumanii*, *Enterococcus faecium*, *Pseudomonas aeruginosa*, which are resistant to at least one antimicrobial agent in three or more antimicrobial categories were considered as MDROs [13].

Demographic data, immunosuppressive drug regimens and transplant-related clinical features of the patients were compared regarding the presence of at least one episode of UTI. UTI characteristics including related microorganisms and antibiotic resistant patterns were also further analyzed. Risk factors associated with UTI were explored.

### Statistical methods

The characteristics of the study patients were expressed as mean or median, as appropriate for categorical variables, percentages and variables with continuity. Mann-Whitney U-test and Kruskal-Wallis test were used for comparing median variables between groups and independent samples *t*-test and one-way ANOVA tests were used for comparing parametric variables. For comparing categorical data, Chi-square and Fisher’s exact test was used. Cox regression analysis was used to investigate the risk factors for the development of urinary tract infection. For all statistical analysis, *p* value <0.05 was considered significant. All data are analyzed with SPSS (version 20.0; SPSS Inc, Chicago, IL) statistical package.

## Results

A total of 102 kidney transplant recipients were analyzed in this study. Forty-nine were women (48%) and 53 (52%) were men, with a mean age of 37,6± 12,2 years. The etiologic distribution of underlying primary kidney disease was as follows: primary glomerulonephritis (29/28.4%), autosomal dominant polycystic kidney disease (ADPKD) (6/5.8%), diabetic nephropathy (9/8.8%), hypertensive nephropathy (12/11.7%), others (13/12.7%), and unknown etiology (33/ 32.3%). Five patients had undergone second kidney transplantation. Seventy- seven (75.5%) patients were on hemodialysis, 4 (3.9%) were on peritoneal dialysis (PD), 7 patients were treated consecutively with both HD and PD (6.8%) before the transplantation and 14 (13.7%) patients underwent preemptive transplantation. ATG was used in 33 patients (32.4%), whereas basiliximab was used in 69 patients (67.6%). Almost all patients (n = 101) received calcineurin inhibitors (CNI) (93 patients were on tacrolimus, 7 patients were on cyclosporine). Mammalian target of rapamycin (mTor) inhibitors was prescribed in 9 patients (8.8%) instead of MMF. mTor inhibitor without CNI (1%) was used in only one patient due to CNI induced thrombotic microangiopathy. All patients received corticosteroids. All patients completed their TMP/SMX prophylaxis during the first 6 months without adverse events.

21 patients experienced at least one UTI over the study period and the incidence rate for a UTI was 20.5% across the whole cohort. A total of 67 UTI episodes were analyzed in the UTI group and the mean number of UTIs per person in this group was 3,19±1,8 [Range 1–7]. The median time to the first UTI attack was calculated as 62 days [range 11–205 days] after transplantation. Presentations of 36 (53.7%) UTI episodes were LUTI, 23 (34.3%) were CUTI and 8 (11.9%) were ABU. Three patients (14,2%) experienced UTI-related bacteremia. Forty-seven episodes (70,1%) were seen within the first six months of transplantation. The demographics and clinical characteristics of patients with and without UTI are shown in Table 1. In univariate analysis, age, dialysis vintage, foley catheter dwell time and presence of urologic complications were significantly different between the groups [Table 1]. In multivariate analysis age (p = 0.004; OR 1.106, 95% Confidence Interval [CI]: 1.032–1.184), longer indwelling urinary catheter stay time (p = 0.039; OR 1.297, 95% Confidence Interval [CI]: 1.013–1.661) and urologic complications (p = 0.006; OR 0.190, 95% Confidence Interval [CI]: 0.001–0.320) were found as the main independent risk factors for UTI development in the first year of transplantation [Table 2].

**Table 1:** Demographic and clinical data in patients with or without Urinary Tract Infections (UTI).

	UTI (+) n = 21	UTI (-) N = 81	*p* value
Age (years)	45,6±13,1	35,5±11,1	0.003*
Sex, n (%)			0.085
Female	14 (66.6%)	35 (43.2%)	
Male	7 (33.3%)	46 (56.8%)	
Dialysis vintage (median, months)	8	45	0.002*
Previous transplantation, n (%)	0 (0%)	5 (6.1%)	0.581
Preemptive transplantation, n (%)	2 (9.5%)	12 (14.8%)	0.729
Living, n (%)	3 (14.2%)	19 (23.4%)	0.553
Donor sex (female), n (%)	12 (57.1%)	52 (64.1%)	0.551
Donor age (years)	47,8±10,9	45±11,3	0.325
Induction, n (%)			0.118
ATG	10 (47.6%)	23 (28.4%)	
Basiliximab	11 (52.4%)	58 (71.6%)	
Maintenance immunosuppressive drugs			0.090
Tacrolimus	18 (85.7%)	67 (82.7%)	
Cyclosporine	2 (9.5%)	5 (23.8%)	
Tacrolimus+mTor inhibitors	0 (0%)	9 (11.1%)	
mTor inhibitors	1 (4.8%)	0 (0%)	
DM or PTDM, n (%)	8 (38%)	23 (28.4%)		0.430
Hospitalization duration at transplantation (days)	12,8±9,8	8,9±3,2		0.091
Urinary catheter dwell time (days)	10,9±7,3	6,9±1,4	0.02*
Double J stent (days)	64,1±27,1	53,4±16,4	0.096
Urologic complications after transplantation, n (%)	6 (28.6%)	2 (2.5%)	0.001*
DGF, n (%)	5 (23.8%)	7 (8.6%)	0.120
Rejection, n (%)	7 (33.3%)	19 (23.5%)	0.403
Immunosuppressive drug			
Trough levels (ng/mL)			
Tacrolimus	8,6±0,7	8,24±1,6	0.132
Cyclosporin	126±11,3	140±23,4	0.472
mTor inhibitor	7,3±0	5,2±1,5	0.400
Creatinine at 1^st^ month (mg/dL)	1,3±0,8	1,25±0,4	0.623
Creatinine at 1^st^ year (mg/dL)	1,21±0,6	1,12±0,4	0.977
eGFR at 1^st^ month (ml/min)	63.4±27.4	67.6±20.6	0.169
eGFR at 1^st^ year (ml/min)	63.6±27.3	70±20	0.352
eGFR difference at 1^st^ year (ml/min) (median, IQR)	1.78 [–6,2–12,7]	2.06 [–7–15,4]	0.960

ATG, anti-thymocyte globulin; mTor, mammalian target of rapamycin; DM, diabetes mellitus; PTDM, post-transplant diabetes mellitus; DGF, delayed graft function; GFR, glomerular filtration rate

*p<0.05, statistically significant.

**Table 2:** Independent risk factors for urinary tract infections in the first year after transplantation.

Parameters	OR (95% CI)	*p value*
Age	1.106 (1.032–1.184)	0.004*
Sex (female)	0.163 (0.023–1.142)	0.068
Dialysis vintage	0.994 (0.980–1.007)	0.346
Urinary catheter dwell time	1.297 (1.013–1.661)	0.039*
Double J stent duration	1.030(0.976–1.087)	0.287
DGF	2.878 (0.362–22.850)	0.317
Urologic complications after transplantation	0.190 (0.001–0.320)	0.006*

DGF, delayed graft function

*p<0.05, statistically significant.

The most common pathogen was *Escherichia coli*, isolated in 52 episodes (73,2%), followed by *Klebsiella pneumoniae* in 12 (16,9%) and fungal species were found as causative agents in 3 (4,2%) UTI episodes. Methicillin-resistant *Staphylococcus epidermidis* was isolated in one and *Enterococcus faecalis* was isolated in three UTI episodes. In three UTI episodes, two different microorganisms were isolated. In total, 71 microorganisms were isolated in all patients. After excluding fungal causes, 43 out of 68 bacterial microorganisms (63.2%) were caused by ESBL producing microorganisms. In total 46 (67.6%) isolates were identified as MDROs. In antibiotic susceptibility tests, the majority of *Escherichia coli* and *Klebsiella pneumoniae*, 84.6% and 83.3% respectively, were resistant to TMP-SMX. Of the 52 *Escherichia coli* isolates, 78.8% were resistant to the quinolones. In *Klebsiella pneumoniae* isolates, 25% were found to be quinolone resistant. No bacteria were found to be carbapenem-resistant. Therefore, 49.2% were treated with carbapenems in our cohort. The mean duration of treatment was 9,2±4 days. A detail on UTI attacks, including causative agents, classes of antibiotic used and antibiotic-resistant patterns of the most common bacteria are summarized in Tables 3 and 4.

**Table 3:** Causative agents and treatment choices for UTI during the first year of transplantation (71 agents in total 67 UTI attack).

Microorganisms	n, (%)
*Escherichia coli*	52 (73.2%)
ESBL (+)	33 (63.4%)
*Klebsiella pneumoniae*	12 (16.9%)
ESBL (+)	10 (83.3%)
Enterococcus faecalis*	3 (4.2%)
*Staphylococcus epidermidis*	1 (1.4%)
*Candida* spp.	3 (4.2%)
Treatments**	UTI attack n, (%)
Penicillins	2 (3%)
Cephalosporins	11 (16.4%)
Fosfomycin	11 (16.4%)
Ciprofloxacin	2 (3%)
Nitrofurantoin	4 (6%)
Carbapenems	33 (49.2%)
Antifungals	3 (4.4%)
No treatment***	5 87.5%)

*isolated with other bacteria

** four patients were treated with two class of antibiotics

***patients with asymptomatic bacteriuria.

ESBL, Extended spectrum beta-lactamase.

**Table 4:** Antibiotic resistance patterns of the most common isolated bacteria.

	*Escherichia coli (n = 52)*	*Klebsiella pneumoniae* (n = 12)
Quinolones, n (%)	41 (78.8%)	3 (25%)
Penicillins, n (%)	37 (71.1%)	11 (91.7%)
Penicillins/beta lactamases, n (%)	18 (34.6%)	6 (50%)
Cephalosporins, 1st or 2nd generation, n (%)	30 (57.7%)	9 (75%)
Cephalosporins, 3rd generation, n (%)	28 (53.8%)	9 (75%)
Fosfomycin, n (%)	7 (13.5%)	3 (25%)
Nitrofurantoin, n (%)	3 (5.8%)	4 (33.3%)
Aminoglycosides, n (%)	6 (11.5%)	1 (8.3%)
Trimethoprim-sulfamethoxazole, n (%)	44 (84.6%)	10 (83.3%)

Fifteen patients (71.4%) (7 male/8 female) had at least one MDROs related UTI. Among all UTI patients, 12 patients (57.1%) had recurrent UTI attacks while 6 of them (50%) had recurrent UTI with MDROs. When we examined UTI episodes in detail, it was noteworthy to discover that male patients were more often infected with MDROs (32 vs. 14 episodes, p<0.001) and had more recurrent UTIs when compared to female patients. The male recipients also had more CUTI at presentation compared with female recipients (Table 5).

**Table 5:** Comparison of male and female patients with UTI.

	Male (n = 7)	Female (n = 14)	*P value*
Age (years)	46,5±11,5	45,1±14,2	0.808
Dialysis vintage (months) (median)	60	42	0.930
Living/Deceased (n)	6 (85.7%)	12 (85.7%)	0.659
Induction (ATG) (n) (%)	4 (57.1%)	6 (42.8)	0.536
DM or PTDM (n) (%)	4 (57.1%)	4 (28.6)	0.346
Double J stent (days)	60,7±17	65,8±31,4	0.633
Indwelling urinary catheter (days)	13,3±6,8	9,7±7,5	0.056
Urologic complication (n) (%)	3 (42.8%)	3 (21.4%)	0.354
Total UTI episodes (n)	34	33	0.108
Mean UTI episodes (n)	4,85	2,35	0.002*
Clinical Presentations			0.005*
(n) (%)			
CUTI	15 (44.1%)	8 (24.2%)	
LUTI	12 (35.3%)	24 (72.7%)	
ABU	7 (20.6%)	1 (3%)	
Microorganisms (n)			0.505
Bacteria	36	32	
Fungal	1	2	
MDROs (episodes) (n) (%)	32(88.9%)	14 (43.7%)	<0.001*
Patients with recurrent UTI episodes (n) (%)	6 (85.7%)	6 (42.8%)	**0.159**
Patients with recurrent UTI episodes with MDRO (n) (%)	5 (71.4%)	1 (7.1)%	0.006*

DM, diabetes mellitus; PTDM, post-transplant diabetes mellitus; ATG, anti-thymocyte globulin.

*p<0.05, statistically significant.

In the analysis of fungal UTI episodes, all of them were resistant species and they have not accepted colonization even if they occurred while the patients still had ureteral stents. All three patients required treatment with voriconazole or anidulafungin and all were treated successfully.

In regard to immunosuppressive regimes, induction regimens were not different between the patients with UTI and those without UTI. Maintenance immunosuppressive drugs and the trough levels of the drugs were similar. Also, when comparing the patients regarding those with UTI caused by MDROs and not, there were no significant differences between the groups. Immunosuppressive drugs and drug levels were also similar in males and females. Furthermore, there was no association between acute rejection episodes and UTI development. There was no impact of UTI even in recurrent patients, on short-term graft functions regarding creatinine and GFR levels.

## Discussion

Post-transplant UTIs in kidney transplant patients are important causes of acute graft dysfunction. Increased morbidity and hospitalization rates are further major consequences of UTIs in kidney transplant recipients. Therefore understanding and exploring the UTI details is very crucial in transplant practice. In our study we found that UTI incidence in the first year after transplantation was 20.5%. Although the incidence of UTI in kidney transplant recipients has been reported ranging between 7%-80% depending on the diagnostic criteria used, our UTI rate is relatively lower than reported in other studies where the first year UTIs are evaluated [1, 4, 14, 15]. In our study a lower rate of asymptomatic bacteriuria was also found. The explanation for the lower rate of ABU may be that a regular screening program was not used and urine culture studies were only performed in patients with UTI symptoms or pyuria in urinalysis observed during regular visits or before urologic invasive procedures. Most patients with UTI attack presented as LUTI. CUTI incidence was found to be 34.3%. Only three attacks of CUTI progressed to bacteremia. The relatively mild course of UTI attacks might result from early detection and immediate antibiotic treatment in our cohort.

It has previously been reported that, early (<3 weeks after transplantation) ureteric stent removal is associated with a lower rate of UTIs [16, 17]. In our center we have not followed this approach. Even though, presence of longer duration of ureteral stent was found, we did not show a higher rate of UTIs in our cohort. Other factors such as, age, a longer duration of indwelling urinary catheter and presence of urologic complications, stood out as important risk factors in our patients. Thus, we recommend that patients with risk factors should be carefully screened for LUTI, CUTI and ABU in the first year after transplantation.

The rates of UTI caused by ESBL-producing organisms were high in our cohort. More than half of UTI patients were exposed to at least one episode of UTI with MDROs. According to a recently published study from Australia, the authors isolated an ESBL-producing organisms in only 3,9% of urine cultures [18]. Bodro et. al. demonstrated that 37% of bacteria considered as MDROs in kidney transplant recipients’ UTIs [10]. Nevertheless, there are some studies showing higher rates of UTI with MDROs in kidney transplant recipients similar to our findings [19, 20]. It is generally accepted that the prevalence of UTI caused by MDRO varies from country to country. According to a recently published meta-analysis, more than 50% of isolates causing UTI in kidney transplant recipients were shown to be resistant to more than 50% of the antibiotics used in Middle East countries [21]. Moreover, in another paper from the US published in 2020, 58.5% of the recipients with UTI had at least one episode with MDROs [22]. Thus we can conclude that the increasing rate of MDROs in transplant recipients may be a major concern worldwide in the coming years.

Our results gave us the opportunity to explore our infection control methods and came to a conclusion that, prophylactic use of quinolones up to seven days postoperatively may be responsible from the high rate of UTIs with MDROs [11]. In 2011, Rafat et al. studied the efficacy of extended prophylaxis with oflaxacin in kidney transplant recipients and they found decreased rate of UTI in these population. However they also discuss that emergence of resistant organisms is the major concern of this approach [23]. Indeed, we believe that following this approach was the major reason for high resistant strain rate in our patients. On antibiotic susceptibility tests, quinolone resistance rate was found to be 64.7%, which is higher than previous studies [21, 22]. Greismann S et al showed that quinolone resistance rate was about 47% even though they did not use quinolones for prophylaxis [22]. In the study published by Behzad D et al, meta-analysis of the antibiotic resistance for gram-negative bacteria suggests that the ciprofloxacin resistance rate was 53.1% [21]. Therefore, changing our method of prophylaxis was the most important consequence of this study. We stopped prophylactic use of quinolones in our practice in line with study results and recent recommendations that advised the use of single-dose cephalosporin [24, 25]. There are also some concerns on prophylactic TMP/ SMX use for the prevention of *Pneumocystis jirovecii*, since TMP/SMX use may eventuate in increasing antimicrobial resistance especially among uropathogenic microorganisms (22). Singh et al. reported administration of TMP/SMX prophylaxis was associated with the significant rise in TMP/SMX resistance rate when comparing kidney transplant recipients without TMP/SMX prophylaxis (89% vs. 48%) [26]. In our cohort, almost 85% of the isolated bacteria were identified as TMP/SMX resistant. Since the use of TMP/SMX for *Pneumocystis jirovecii* prophylaxis cannot be avoided, it is important to control other risk factors such as unnecessary antibiotic use before and after transplantation.

There are many studies showing that urinary tract infections are more common in female recipients [1, 4, 5]. However, there are also some studies that did not find any difference with respect to gender [7, 22, 27–29]. Interestingly, in our study, we showed that recurrent urinary tract infections with resistant microorganisms are more common in male transplant recipients. Male recipients also presented more often with CUTI. Therefore, our data suggest, starting treatment with a wide-spectrum antibiotic may be warranted for UTI infections in male transplants since they tend to be caused by resistant microorganisms and have a tendency to recur. There are studies in both the general population and the transplant population to support these findings [30–33]. Urinary outflow obstruction due to prostate, possible prostatitis, and inadequate response to antibiotics due to long uroepithelial tissue in male recipients compared to females are the mechanisms that explain this situation.

It is important to emphasize that, a UTI caused by ESBL-producing microorganisms carries an almost three times greater risk of recurrence [6]. Brakemeir et al reported that the recurrence rate of UTI with ESBL producing bacteria was found to be 54% [29]. Our findings were also consistent with this previously published data.

These results should be interpreted with caution due to the single-center and retrospective nature of the study and the relatively small number of patients. The low number of events limits further statistical analysis for exploring the exact effect of male gender on resistant and recurrent UTIs.

## Conclusion

Our results suggested that advanced age, longer duration of bladder catheterization and urologic complications are the risk factors for UTI in the first year. Although a relatively low incidence of UTI was seen in our cohort, study results represent the growing incidence of UTI with MDROs in KT recipients. Restriction of unnecessary antibiotic use is crucial to mitigate resistant UTIs. Since there is a high proportion of UTI caused by resistant microorganisms in male transplant patients, infection control methods should be applied even more vigorously especially in male transplant patients. This study also highlights the importance of monitoring causative agents of UTIs in order to improve empiric therapy in transplant practice.

Our results shed an interesting perspective on UTI in kidney transplant recipients. We strongly believe that each transplant center should explore their own UTI risk factors and causative agents that will direct them to manage these patients correctly.

## References

[1] Goldman JD, Julian K. Urinary tract infections in solid organ transplant recipients: Guidelines from the American Society of Transplantation Infectious Diseases Community of Practice. Clin Transplant. 2019; 33(9):e13507. 10.1111/ctr.13507 30793386

[2] Bodro M, Linares L, Chiang D, Moreno A, Cervera C. Managing recurrent urinary tract infections in kidney transplant patients. Expert Rev Anti Infect Ther. 2018; 16(9):723–732. 10.1080/14787210.2018.1509708 30092153

[3] Kotagiri P, Chembolli D, Ryan J, Hughes PD, Toussaint ND. Urinary Tract Infections in the First Year Post-Kidney Transplantation: Potential Benefits of Treating Asymptomatic Bacteriuria. Transplant Proc. 2017; 49(9):2070–2075. 10.1016/j.transproceed.2017.07.008 29149963

[4] Ariza‐Heredia EJ, Beam EN, Lesnick TG, Kremers WK, Cosio FG, Razonable RR. Urinary tract infections in kidney transplant recipients: role of gender, urologic abnormalities, and antimicrobial prophylaxis. Ann Transplant. 2013; 18: 195–204 10.12659/AOT.883901 23792521

[5] Gozdowska J, Czerwińska M, Chabros Ł, et al. Urinary tract infections in kidney transplant recipients hospitalized at a transplantation and nephrology ward: 1‐year follow‐up. Transplant Proc. 2016;48(5):1580–1589 10.1016/j.transproceed.2016.01.061 27496451

[6] Alevizakos Michail, Nasioudis Dimitrios, Mylonakis Eleftherios. Urinary Tract Infections Caused by ESBL-producing Enterobacteriaceae in Renal Transplant Recipients: A Systematic Review and Meta-Analysis. Transpl Infect Dis. 2017;19(6). 10.1111/tid.12759 28803446

[7] Vidal E, Torre-Cisneros J, Blanes M, Montejo M, Cervera C, Aguado JM, et al; Spanish Network for Research in Infectious Diseases (REIPI). Bacterial urinary tract infection after solid organ transplantation in the RESITRA cohort. Transpl Infect Dis. 2012;14(6):595–603 10.1111/j.1399-3062.2012.00744.x 22650416

[8] Parasuraman R, Julian K; AST Infectious Diseases Community of Practice. Urinary tract infections in solid organ transplantation. Am J Transplant. 2013;13 Suppl 4:327–36. 10.1111/ajt.12124 23465025

[9] Pinheiro HS, Mituiassu AM, Carminatti M, Braga AM, Bastos MG. Urinary tract infection caused by extended‐spectrum beta‐lactamase‐ producing bacteria in kidney transplant patients. Transplant Proc. 2010; 42(2):486–487. 10.1016/j.transproceed.2010.02.002 20304172

[10] Bodro M, Sanclemente G, Lipperheide I, et al. Impact of antibiotic resistance on the development of recurrent and relapsing symptomatic urinary tract infection in kidney recipients. Am J Transplant. 2015;15(4):1021–1027. 10.1111/ajt.13075 25676738

[11] Delmas-Frenette C, Dorais M, Tavares-Brum A, et al. Epidemiology and outcome of antimicrobial resistance to gram-negative pathogens in bacteriuric kidney transplant recipients. Transpl Infect Dis. 2017; 19(4): 10.1111/tid.12722. 10.1111/tid.12722 28486744

[12] Britt NS, Hagopian JC, Brennan DC, et al. Effects of recurrent urinary tract infections on graft and patient outcomes after kidney transplantation. Nephrol Dial Transplant. 2017;32(10):1758–1766. 10.1093/ndt/gfx237 28967964

[13] Magiorakos AP, Srinivasan A, Carey RB et al. Multidrug-resistant, extensively drug-resistant and pandrug-resistant bacteria: an international expert proposal for interim standard definitions for acquired resistance. Clin. Microbiol. Infect. 2012; 18: 268–81. 10.1111/j.1469-0691.2011.03570.x 21793988

[14] Naik AS, Dharnidharka VR, Schnitzler MA, et al. Clinical and economic consequences of first-year urinary tract infections, sepsis, and pneumonia in contemporary kidney transplantation practice. Transpl Int. 2016;29(2):241–252. 10.1111/tri.12711 26563524 PMC4805426

[15] Gołębiewska JE, Dębska-Ślizień A, Rutkowski B. Urinary tract infections during the first year after renal transplantation: one center’s experience and a review of the literature. Clin Transplant. 2014;28(11):1263–1270. 10.1111/ctr.12465 25251447

[16] Visser IJ, van der Staaij JPT, Muthusamy A, Willicombe M, Lafranca JA, Dor FJMF. Timing of Ureteric Stent Removal and Occurrence of Urological Complications after Kidney Transplantation: A Systematic Review and Meta-Analysis. J Clin Med. 2019;8(5):689. 10.3390/jcm8050689 31100847 PMC6572676

[17] Cai JF, Wang W, Hao W, et al. Meta-analysis of Early Versus Late Ureteric Stent Removal After Kidney Transplantation. Transplant Proc. 2018;50(10):3411–3415. 10.1016/j.transproceed.2018.08.033 30577214

[18] Olenski S, Scuderi C, Choo A, et al. Urinary tract infections in renal transplant recipients at a quaternary care centre in Australia. BMC Nephrol. 2019;20(1):479. 10.1186/s12882-019-1666-6 31881863 PMC6935183

[19] Kiros T, Asrat D, Ayenew Z, Tsige E. Bacterial urinary tract infection among adult renal transplant recipients at St. Paul’s hospital millennium medical college, Addis Ababa, Ethiopia. BMC Nephrol. 2019;20(1):289. 10.1186/s12882-019-1485-9 31366333 PMC6668100

[20] Yuan X, Liu T, Wu D, Wan Q. Epidemiology, susceptibility, and risk factors for acquisition of MDR/XDR gram-negative bacteria among kidney transplant recipients with urinary tract infections. Infect Drug Resist. 2018;14(11):707–15. 10.2147/IDR.S163979 PMC5957067 29785131

[21] Behzad D, Hakimeh A, Hossein R, Khaledi A. A middle east systematic review and meta-analysis of bacterial urinary tract infection among renal transplant recipients; Causative microorganisms. Microb Pathog. 2020 11;148:104458. 10.1016/j.micpath.2020.104458 32835776

[22] Greissman S, Mattiazzi A, Mendoza M, Natori Y, Grady M, Quinonez J, et al. Antimicrobial resistance and recurrent bacterial urinary tract infections in hospitalized patients following kidney transplantation: A single-center experience. Transpl Infect Dis. 2020 8; 22(4):e13337. 10.1111/tid.13337 32452596

[23] Rafat C, Vimont S, Ancel PY, Xu-Dubois YC, Mesnard L, Ouali N, et al. Ofloxacin: new applications for the prevention of urinary tract infections in renal graft recipients. Transpl Infect Dis. 2011 8;13(4):344–52. 10.1111/j.1399-3062.2011.00602.x 21299776

[24] Nishimura S, Wada K, Araki M, Sadahira T, Maruyama Y, Mitsui Y, et al. Use of single-dose perioperative antimicrobial therapy is acceptable in recipients of living-donor renal transplants in the rituximab era. J Infect Chemother. 2019 4; 25(4): 247–252. 10.1016/j.jiac.2018.11.013 30583958

[25] Orlando G., et al. One-shot versus multidose perioperative antibiotic prophylaxis after kidney transplantation: a randomized, controlled clinical trial. Surgery, 2015. 157: 104. 10.1016/j.surg.2014.06.007 25304836

[26] Singh R, Bemelman FJ, Hodiamont CJ, Idu MM, Ten Berge IJ, Geerlings SE. The impact of trimethoprim-sulfamethoxazole as Pneumocystis jiroveci pneumonia prophylaxis on the occurrence of asymptomatic bacteriuria and urinary tract infections among renal allograft recipients: a retrospective before-after study. BMC Infect Dis. 2016;16:90. 10.1186/s12879-016-1432-3 26912326 PMC4766656

[27] Dantas SR, Kuboyama RH, Mazzali M, Moretti ML. Nosocomial infections in renal transplant patients: risk factors and treatment implications associated with urinary tract and surgical site infections. J Hosp Infect. 2006; 63(2):117–123. 10.1016/j.jhin.2005.10.018 16517007

[28] Bodro M, Sanclemente G, Lipperheide I, et al. Impact of urinary tract infections on short-term kidney graft outcome. Clin Microbiol Infect. 2015; 21(12):1104.e1–1104.e11048. 10.1016/j.cmi.2015.07.019 26235196

[29] Brakemeier S, Taxeidi SI, Zukunft B, et al. Extended-Spectrum Beta-Lactamase-Producing Enterobacteriaceae-Related Urinary Tract Infection in Kidney Transplant Recipients: Risk Factors, Treatment, and Long-Term Outcome. Transplant Proc. 2017;49(8):1757–1765. 10.1016/j.transproceed.2017.06.033 28923621

[30] Pilmis B, Scemla A, Join-Lambert O, et al. ESBL-producing enterobacteriaceae-related urinary tract infections in kidney transplant recipients: incidence and risk factors for recurrence. Infect Dis (Lond). 2015;47(10):714–718. 10.3109/23744235.2015.1051107 26024285

[31] Bischoff S, Walter T, Gerigk M, Ebert M, Vogelmann R. Empiric antibiotic therapy in urinary tract infection in patients with risk factors for antibiotic resistance in a German emergency department. BMC Infect Dis. 2018;18(1):56. 10.1186/s12879-018-2960-9 29373965 PMC5787273

[32] Almomani BA, Hayajneh WA, Ayoub AM, Ababneh MA, Al Momani MA. Clinical patterns, epidemiology and risk factors of community-acquired urinary tract infection caused by extended-spectrum beta-lactamase producers: a prospective hospital case-control study. Infection. 2018; 46(4):495–501. 10.1007/s15010-018-1148-y 29748840

[33] Briongos-Figuero LS, Gómez-Traveso T, Bachiller-Luque P, et al. Epidemiology, risk factors and comorbidity for urinary tract infections caused by extended-spectrum beta-lactamase (ESBL)-producing enterobacteria. Int J Clin Pract. 2012; 66(9):891–896. 10.1111/j.1742-1241.2012.02991.x 22897466

